# Augmented reality for ultrasound-guided venipuncture: a pilot study on a simulated vascular access model

**DOI:** 10.1007/s10877-026-01437-9

**Published:** 2026-04-15

**Authors:** Cosimo Aliani, Alberto Morelli, Simona Cristina Aiello, Fulvio Pinelli, Gianluca Villa, Stefano Romagnoli, Leonardo Bocchi

**Affiliations:** 1 Department of Information Engineering, University of Florence, Via di Santa Marta 3, Florence, 50139 Italy; 2 Department of Health Sciences, University of Florence, Viale Pieraccini 6, Florence, 50139 Italy; 3 Vascular Access Center, Azienda Ospedaliera Universitaria Careggi, Florence, 50139 Italy

**Keywords:** Augmented reality, Head-mounted display, Ultrasound-guided venipuncture, Vascular access

## Abstract

To evaluate the feasibility of a low-cost augmented reality (AR) workflow for ultrasound-guided venipuncture and whether AR visualisation is non-inferior to conventional monitor guidance in total procedure time while improving user experience. Real-time ultrasound video from a conventional scanner was captured via an HDMI-to-USB device, pre-processed with a Python/OpenCV pipeline, and streamed over Wi-Fi to a HoloLens 2 headset using WebSocket. A turkey-thigh model with an embedded fluid-filled catheter simulated a peripheral vein. One experienced implantation nurse performed 58 venipuncture attempts with standard monitor guidance and 58 with AR (monitor blanked during AR). Total procedure time (TPT) was recorded for each attempt, and a questionnaire assessed perceived difficulty, image quality, usability, reliability, and ergonomic comfort. With all data included, overall TPT was comparable between standard and AR guidance ($21.32 \pm 7.90$ s vs. $21.64 \pm 10.30$ s; $p = 0.845$). After excluding predefined outliers, overall TPT remained non-significantly lower with AR ($21.40 \pm 6.78$ s vs. $19.90 \pm 6.34$ s; $p = 0.193$); on day 2 the *p*-value decreased to 0.103 ($20.45 \pm 6.32$ s vs. $18.30 \pm 5.12$ s). Questionnaire responses favoured AR for gaze continuity, perceived coordination, usability, and ergonomic comfort, with acceptable image quality. AR-assisted ultrasound-guided venipuncture was feasible and did not add measurable temporal overhead for an expert operator, while improving perceived ergonomics. Larger, multi-operator clinical studies are needed to confirm performance effects and clinical impact.

## Introduction

Ultrasound (US) guidance is a standard of care for several vascular access procedures [3, 4] and is increasingly adopted for peripheral venous cannulation in patients with difficult intravenous access (DIVA) [6]. Compared with landmark-based or blind techniques, US guidance can improve cannulation success and reduce complications, but it remains a highly operator-dependent skill that requires efficient visuomotor coordination.

A common practical limitation of US-guided venipuncture is the reliance on a conventional monitor that may be physically separated from the puncture site. This often forces repeated gaze shifts between the screen and the patient, introducing visual–motor dissociation and increasing cognitive load, especially in stressful environments or for less experienced operators [2, 7, 10]. Maintaining a stable mapping between a two-dimensional US image and three-dimensional anatomy is therefore a key challenge in ultrasound-guided interventions.

Augmented reality (AR) head-mounted displays (HMDs) have been proposed to visualise live US images within the operator’s line of sight, with the goal of improving gaze continuity and cognitive ergonomics during image-guided tasks [5, 8, 10]. Recent studies have also explored AR visualisation specifically for US-guided vascular access, reporting procedural performance comparable to standard monitor guidance together with favourable usability and ergonomic feedback [9, 11]. However, practical and low-complexity integration workflows that can be readily interfaced with conventional ultrasound systems remain less documented.

In this study, we developed a low-cost AR system that streams real-time ultrasound images from a conventional ultrasound machine to a HoloLens 2 headset using a Python/OpenCV and WebSocket-based pipeline. We evaluated the system in a simulated venipuncture setting using a turkey-thigh vascular access model. An expert nurse performed ultrasound-guided venipuncture under conventional monitor guidance and under AR guidance (with the monitor blanked during AR trials). We compared total procedure time and collected structured subjective feedback on usability and ergonomics. We hypothesised that AR visualisation would be non-inferior to conventional monitor guidance in procedural efficiency while improving gaze continuity and perceived ergonomic comfort.

## Materials and methods

This section describes the hardware and software architecture used to stream ultrasound images to the augmented reality headset, the protocol adopted to compare conventional and AR-guided venipuncture, and the statistical methods employed to analyse the collected data.

### System architecture

A custom software pipeline was developed in Python to acquire, process, and transmit real-time ultrasound (US) video to a head-mounted augmented reality (AR) display.

A conventional medical ultrasound device was routed via HDMI to an Elgato CamLink 4 K (Elgato, Corsair GmbH, Munich, Germany) capture device, which converted the video signal to USB for acquisition on a laptop computer. Video frames were captured and processed in real time using the OpenCV library [1]. The application handled basic pre-processing operations, including resolution adjustment and interactive cropping of the region of interest, and monitored acquisition parameters such as frame rate.

Processed frames were then streamed to the Microsoft HoloLens 2 headset over a local wireless network using the WebSocket protocol. On the headset, the incoming video stream was visualised as an AR overlay within the operator’s field of view, allowing simultaneous direct inspection of the puncture site and the corresponding ultrasound image.

The graphical user interface (GUI), implemented with PySide6, allowed the operator to configure the main acquisition parameters: selection of the video input source, choice of resolution (from 480p to 1080p), activation of video recording, and definition of an interactive cropping window. A built-in frame rate monitor provided feedback on streaming performance.

The ultrasound probe and scanning settings were chosen according to standard practice for vascular access procedures.

### Experimental model

A low-cost, anatomy-like model was created using fresh turkey thighs to simulate human tissues for venipuncture training. Turkey thighs were selected because their echogenicity and layered structure (skin, subcutaneous tissue, muscle) approximate those of human tissue in ultrasound imaging and allow needle insertion with realistic tactile feedback.

**Fig. 1:**
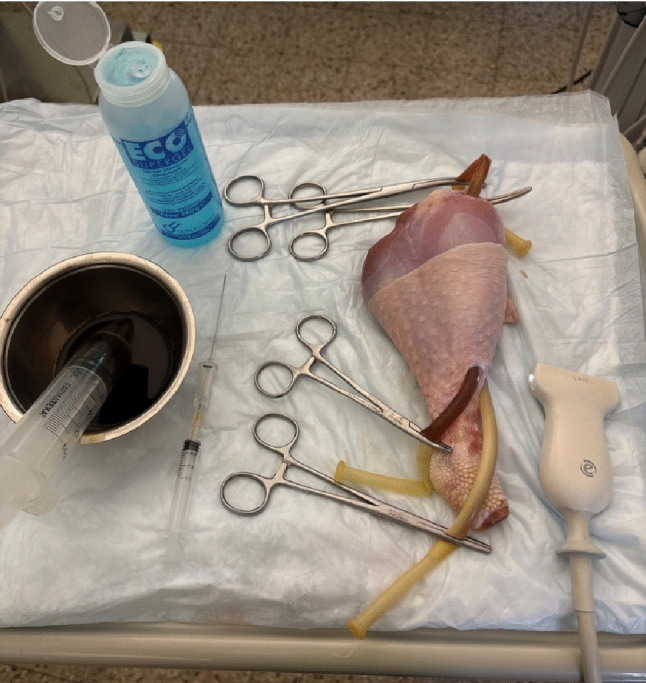
Experimental setup used during the test

To simulate vascular structures, a rubber catheter was inserted through each turkey thigh, passing from side to side and embedded within the soft tissue. The stiffness and internal diameter of the catheter were chosen to approximate those of a deep vein. Once the catheter was in place, its lumen was filled with a coloured iodine-based solution (Betadine), and both ends were clamped to maintain a closed fluid column. Under ultrasound guidance, this fluid-filled catheter appeared as a tubular target that could be easily identified and punctured, mimicking a superficial vein embedded in soft tissue. During repeated puncture attempts, leakage of Betadine through the needle holes progressively reduced the intraluminal pressure and modified the cross-sectional geometry of the catheter. Whenever the lumen appeared partially collapsed or no longer comparable to a physiological superficial vein on ultrasound, the entire specimen (turkey thigh and catheter) was discarded and replaced with a freshly prepared one in order to preserve consistent target characteristics across attempts. The overall arrangement of the model and materials is depicted in Fig. 1.

The turkey thighs were positioned on a dedicated support on the procedure table, reproducing a stable and ergonomically accessible working area for the operator.

### Protocol

This was a single-operator, simulation-based pilot study conducted over two consecutive days at the "Vascular Access Center" of Careggi University Hospital (Florence, Italy). All procedures were performed by an experienced implantation nurse with specific training in ultrasound-guided venipuncture.

On each study day, the operator performed multiple venipuncture attempts on the turkey thigh models under two different visualisation conditions:

1. **Traditional method:** real-time ultrasound guidance using the standard ultrasound monitor;
2. **AR method:** real-time ultrasound guidance using the HoloLens 2 headset, with the US image displayed in the operator’s field of view. During AR-guided attempts, the physical ultrasound monitor was intentionally blanked (black screen) while the video signal continued to be captured by the acquisition system, so that the operator could rely exclusively on the head-mounted display for image visualisation and could not inadvertently refer to the conventional screen.

Across the two experimental days, this resulted in a total of 58 ultrasound-guided venipuncture attempts for each visualisation modality (58 conventional and 58 AR-based).

For each attempt, the operator scanned the model to identify the simulated vessel and then advanced the needle under ultrasound guidance until the vascular target was reached. The same general technique and needle handling strategy were used in both conditions, in order to isolate the effect of the visualisation modality.

The total procedure time (*TPT*) was recorded for every attempt. An attempt was considered successful when the operator achieved stable ultrasound confirmation of needle placement within the Betadine-filled target.

At the end of the study (after completing all attempts in both visualisation conditions over the two experimental days), the operator completed a structured questionnaire evaluating:

- Perceived difficulty of the procedure;
- Perceived image quality;
- Usability and intuitiveness of the visualisation modality;
- Perceived reliability of the system;
- Ergonomic and visual comfort (including gaze continuity and perceived cognitive load).

Responses were collected using closed-ended items with ordered response categories, allowing comparison between the traditional and AR conditions.

### Statistical analysis

Given the pilot nature of the study, no formal *a priori* sample size calculation was performed. All available attempts from the two study days were included in the analysis.

The recorded total procedure time (*TPT*) was analysed using descriptive statistics (mean and standard deviation). The conventional and AR-based venipuncture conditions were then compared using Student’s t-tests to explore the presence of statistically significant differences in procedural times between the two visualisation modalities. All tests were two-sided, with statistical significance set at $\alpha = 0.05$.

Questionnaire responses were analysed descriptively, summarising the distribution of ratings for each item and comparing the patterns between the conventional and AR-based conditions. Qualitative comments provided by the operator were also examined to highlight perceived strengths and weaknesses of the AR system, as well as potential critical issues in its use during ultrasound-guided venipuncture.

## Results

**Fig. 2:**
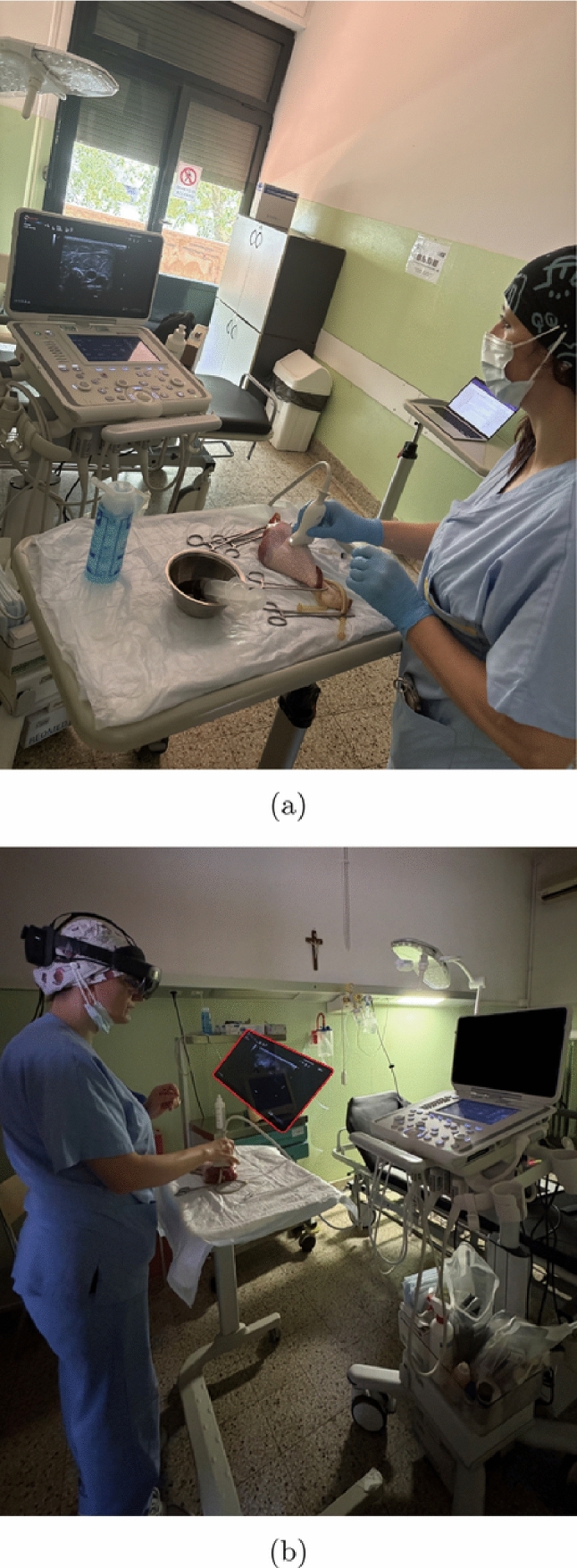
Venipuncture performed using the conventional technique (**a**) and with an augmented-reality (AR) headset (**b**). The virtual display in the lower panel, highlighted in red, was added in post-processing, as it is visible only to the operator through the AR headset

Two photographs captured during the test–the first one using the conventional mode, while the second one using the AR headset–are presented in Fig. 2.

**Fig. 3:**
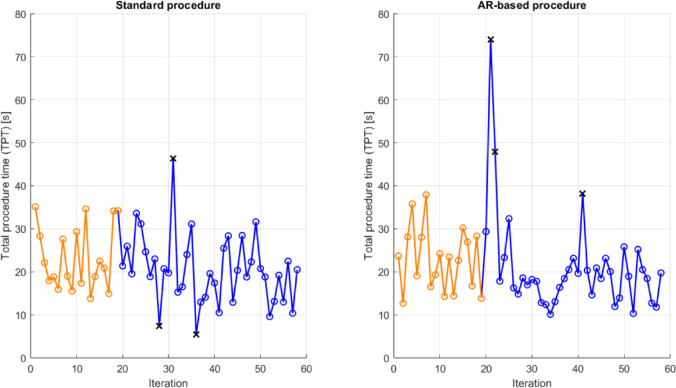
Aggregated *TPT* with the standard procedure (left) and the AR-based procedure (right). In orange are reported data collected during the first day, while in blue are reported data collected during the second day. Time considered outliers are displayed using a black *x*

**Fig. 4:**
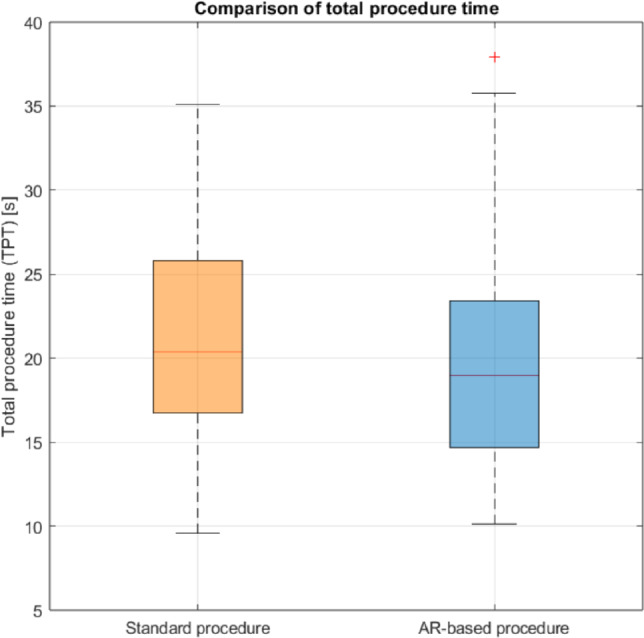
Aggregated *TPT*. The standard procedure is represented in orange, while the AR-based procedure is displayed in blue. Outliers were removed before the computation

Aggregated (first day and second day) total procedure times for both acquisition modalities are shown in Fig. 3. Data points classified as outliers were excluded from the aggregated analysis; in Fig. 3, these outlier measurements are displayed as black *x* markers. A boxplot representation of the total procedure times, computed after excluding outliers, is shown in Fig. 4.

Table 1 reports the mean ± standard deviation of the total procedure time (*TPT*) for the standard and AR-based procedures on day 1, day 2, and across all trials, along with the outcome of paired t-tests.

**Table 1:** Comparison of total procedure time (mean ± standard deviation) between standard and AR-based procedure for day 1, day 2, and all trials combined

Day		Standard TPT [s]	AR-based TPT [s]	*p-value*
1	With outliers	$23.22 \pm 7.41$	$22.95 \pm 7.40$	0.906
	Without outliers	$23.22 \pm 7.41$	$22.95 \pm 7.40$	0.906
2	With outliers	$20.39 \pm 8.06$	$21.00 \pm 11.49$	0.783
	Without outliers	$20.45 \pm 6.32$	$18.30 \pm 5.12$	0.103
1+2	With outliers	$21.32 \pm 7.90$	$21.64 \pm 10.30$	0.845
	Without outliers	$21.40 \pm 6.78$	$19.90 \pm 6.34$	0.193

Symbols: *$p < 0.05$, **$p < 0.01$(paired t-test)

## Discussion and limitations

This pilot study assessed the feasibility of integrating a head-mounted AR display into ultrasound-guided venipuncture using a low-cost streaming pipeline and an anatomy-like vascular access model. Overall, AR-based visualisation achieved procedural times comparable to conventional monitor guidance, suggesting that the proposed workflow can be adopted without introducing a measurable temporal overhead for an expert operator (see Results and Table 1 for quantitative data). A non-significant trend toward lower times on the second day may reflect increasing familiarity with the headset-based visualisation rather than a fundamental performance difference.

From a human factors perspective, comparable procedure time is not unexpected in an expert operator performing a well-consolidated manual task. In such conditions, performance is often limited by needle manipulation and target characteristics rather than by display modality alone. The most relevant added value of AR may therefore lie in cognitive ergonomics: by placing the ultrasound image within the operator’s line of sight, AR can reduce gaze shifts and support visuomotor integration. Consistently, the post-study questionnaire indicated a preference for AR in terms of gaze continuity, perceived coordination, usability, and ergonomic comfort, while maintaining acceptable image quality.

A practical contribution of this work is the demonstration of a simple integration pathway that retrofits a conventional ultrasound machine with AR visualisation using off-the-shelf components. The use of monitor blanking during AR trials was intended to isolate the effect of the head-mounted display and avoid inadvertent reference to the conventional screen, strengthening the internal validity of the comparison in a simulation setting.

Several limitations should be acknowledged. First, this was a single-operator pilot study; results may not generalise to other expert users or to novice operators, who could experience different performance effects and potentially larger benefits from reduced visuomotor dissociation. Second, the evaluation was conducted on a simulated turkey-thigh model, which cannot reproduce clinical variability (patient movement, anatomical heterogeneity, environmental constraints). Third, some technical aspects were not quantitatively characterised in this pilot (e.g., end-to-end latency and frame rate under varying network and lighting conditions), and these parameters may become more relevant in faster manoeuvres or different clinical settings.

Future work should include multi-operator studies, objective endpoints beyond time (e.g., first-attempt success, needle redirections, number of passes), and a systematic technical characterisation of streaming latency and robustness. Ultimately, clinical evaluations will be required to determine whether improved gaze continuity and perceived ergonomics translate into better procedural outcomes, safer performance in difficult access scenarios, or more efficient training pathways.

## Conclusion

This pilot study demonstrated the feasibility of a low-cost augmented reality workflow for ultrasound-guided venipuncture based on real-time ultrasound streaming to a HoloLens 2 headset. In a simulated vascular access model and expert-user setting, AR visualisation achieved procedural performance comparable to conventional monitor-based guidance, supporting the practical integration of the proposed system without an evident time penalty.

Beyond procedural feasibility, the AR configuration showed potential advantages in terms of gaze continuity and perceived ergonomic comfort, which are relevant factors in ultrasound-guided manual tasks. These findings suggest that head-mounted AR visualisation may represent a useful support tool for image-guided venipuncture, particularly from a human-factors perspective.

The present results should be interpreted in light of the pilot design (single operator, simulated model, and limited technical characterisation of streaming performance). Future studies should include multiple operators with different experience levels, objective performance metrics beyond procedure time, and systematic evaluation of latency/robustness, followed by clinical validation in real-world settings.

## Data Availability

No datasets were generated or analysed during the current study.
